# Reversible cerebral vasoconstriction syndrome due to teprotumumab: two case reports

**DOI:** 10.1093/omcr/omae085

**Published:** 2024-08-06

**Authors:** Mohamed Elfil, Pashayar P Lookian, Kanchan Kumari, Mohammad Aladawi, Mark Jedras, Steven M Phillips, Mithun G Sattur

**Affiliations:** Department of Neurological Sciences, University of Nebraska Medical Center, 988440 Nebraska Medical Center, Omaha, NE 68198-8440, United States; Department of Neurosurgery, University of Nebraska Medical Center, 4242 Farnam St., Omaha, NE 68131, United States; Department of Neurological Sciences, University of Nebraska Medical Center, 988440 Nebraska Medical Center, Omaha, NE 68198-8440, United States; Department of Neurological Sciences, University of Nebraska Medical Center, 988440 Nebraska Medical Center, Omaha, NE 68198-8440, United States; Department of Neurological Sciences, University of Nebraska Medical Center, 988440 Nebraska Medical Center, Omaha, NE 68198-8440, United States; Department of Neurological Sciences, University of Nebraska Medical Center, 988440 Nebraska Medical Center, Omaha, NE 68198-8440, United States; Department of Neurosurgery, University of Nebraska Medical Center, 4242 Farnam St., Omaha, NE 68131, United States

**Keywords:** reversible cerebral vasoconstriction syndrome, teprotumumab, monoclonal antibody, thyroid eye disease, graves’ eye disease, subarachnoid hemorrhage, insulin-like growth factor-1

## Abstract

Background: Reversible Cerebral Vasoconstriction Syndrome (RCVS) involves cerebral vasculature constriction and dilation. While the exact pathophysiology of RCVS is still not fully understood, there are multiple etiological factors suggested to be implicated in triggering RCVS. We report two RCVS cases potentially linked to teprotumumab. Case 1: A 59-year-old female with Graves’ eye disease (GED) developed leg weakness and headache after initiating teprotumumab, and neuroimaging studies revealed multifocal cerebral vasospasm (CVS). Verapamil mitigated vasospasm and the patient overall improved. Case 2: A 71-year-old female with GED developed thunderclap headache two months after starting teprotumumab, with subarachnoid hemorrhage (SAH) and CVS revealed on neuroimaging studies. The patient improved on verapamil and was discharged without deficits. Conclusions: The temporal correlation between teprotumumab initiation and RCVS’s symptom onset raises concern for the potential involvement of teprotumumab in triggering RCVS via disrupting cerebrovascular modulation. Further research is needed to investigate this proposed association.

## Introduction

Reversible cerebral vasoconstriction syndrome (RCVS) is characterized by segmental narrowing of the cerebral vasculature which may or may not be accompanied by nonaneurysmal subarachnoid hemorrhage (SAH) [1]. Clinically, RCVS presents with a sudden onset of thunderclap headache which might be associated with nausea, vomiting, photophobia, phonophobia, confusion and/or visual changes [2]. Although the pathophysiology of RCVS is not fully clear, there are a few suggested risk factors including pregnancy [3], metabolic derangements [4], cervical artery dissection, and several medications [3].

Teprotumumab is a monoclonal antibody that inhibits insulin-like growth factor 1 receptor (IGF-1R) and used for treating Graves’ eye disease (GED) [5]. There are a few reported neurological adverse effects of teprotumumab including cognitive impairment [6], and sensorineural hearing loss [7]. To our knowledge, teprotumumab has not been reported in literature to cause RCVS. We present two RCVS cases likely attributed to teprotumumab.

## Case reports

### Case 1

A 59-year-old female with history of GED and chronic back pain, with spinal fusion and hydromorphone pump placement, was transferred to our institution for evaluation of acute bilateral leg weakness and left foot drop accompanied by a severe headache which started suddenly upon waking up. She reported experiencing severe headaches following the initiation of teprotumumab for GED approximately 4–5 months earlier than this admission. Her neurological examination demonstrated severe weakness of left ankle plantar and dorsiflexion, mild weakness of left hip flexion, knee flexion, and knee extension, and loss of vibration in the left foot. The initial laboratory work-up was remarkable only for low calcium level (8.1 mg/dl) and low vitamin B6 level (18.4 nmol/l). Cerebrospinal fluid (CSF) studies were remarkable only for mildly elevated lymphocyte-predominant white blood cell (WBC) count (10/ul). Electromyography and nerve conduction studies were unremarkable for either neuropathy or radiculopathy with decreased activation indicative of a central cause of weakness. Magnetic resonance imaging (MRI) of the brain/spine was not obtained due to hydromorphone pump placement.

**Figure 1:**
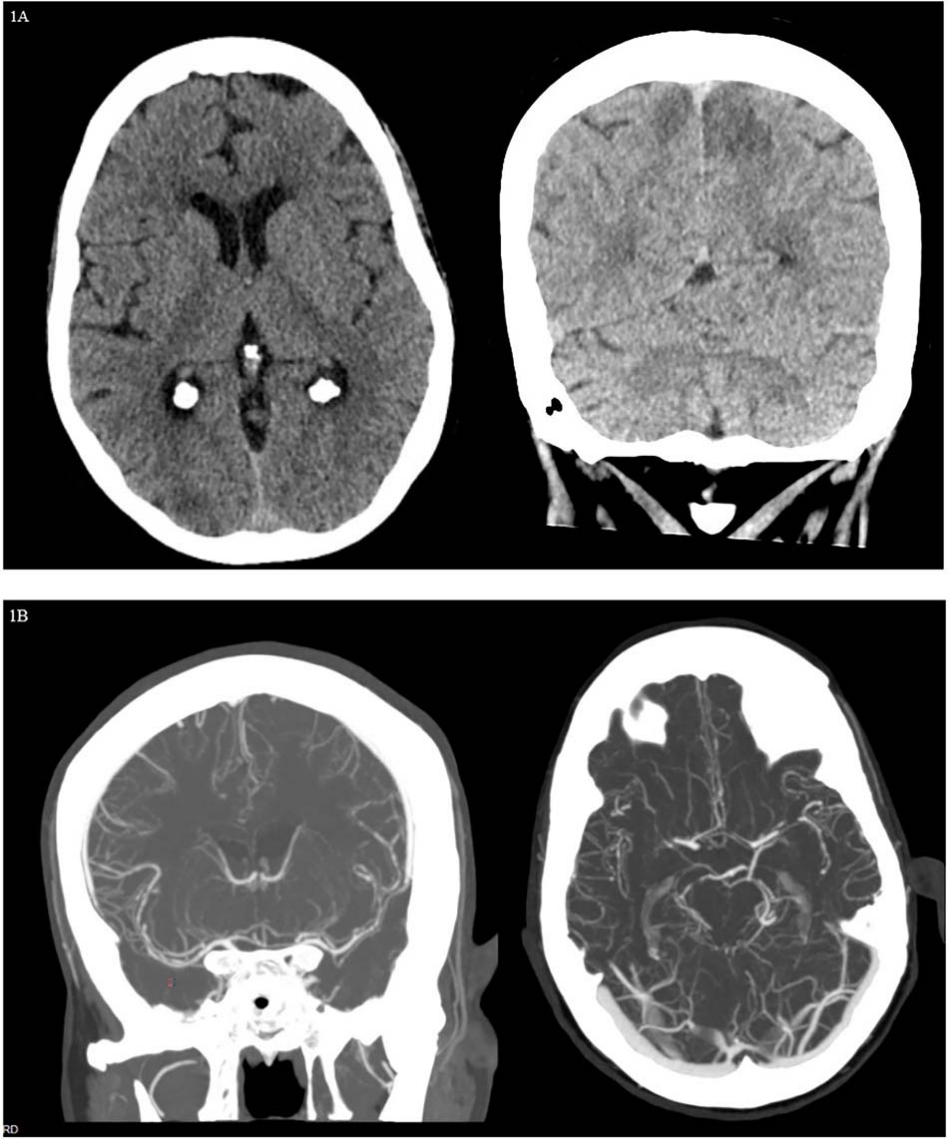
Case 1. CT head and CTA head and neck findings during the first admission. (**A**) CT head showing bilateral hypoattenuation within the paramedian parietal and occipital lobes. (**B**) CTA head and neck showing multifocal stenosis in bilateral MCAs and PCAs, along with bilateral multifocal luminal irregularities in ACAs.

Four days following admission, she reported sudden lower visual field loss. Further examination revealed bilateral left lower quadrantanopsia. Computed tomography (CT) head showed bilateral hypoattenuation within the paramedian parietal and occipital lobes (Fig. 1A). CT angiography (CTA) head and neck showed areas of mild-to-moderate multifocal stenosis of bilateral middle cerebral arteries (MCAs), posterior cerebral arteries (PCAs), and anterior cerebral arteries (ACAs) (Fig. 1B). She was started on verapamil 120 mg nightly for treatment of cerebral vasospasm (CVS) and then discharged to an acute rehabilitation facility (Table 1).

**Table 1:** A summary of the demographic data, clinical presentations, laboratory workup, neuroimaging studies, and clinical courses of the two cases

	Case 1	Case 2
Age & Gender	• 59-year-old female	• 71-year-old female
Timing of neurological presentation after starting Teprotumumab	• 4-5 months after starting teprotumumab	• 2 months after starting teprotumumab
Presenting neurological symptoms (In a chronological order)	• Intermittent severe headaches following teprotumumab initiation • Acute bilateral leg weakness and left foot drop accompanied by severe headache • Four days later, sudden lower visual field loss with bilateral left lower quadrantanopia	• Sudden onset thunderclap headache associated with nausea and vomiting
Neurological examination findings	• Left ankle plantar and dorsiflexion weakness (MRC 0/5) • Mild weakness of left hip flexion, knee flexion, and knee extension (MRC 4/5) • Loss of vibration in left foot • Bilateral left lower quadrantanopia	• Unremarkable
Laboratory findings and ancillary studies	• Unremarkable CBC. • Low calcium (8.1 mg/dl) • Low vitamin B6 (18.4 nmol/l) • Normal folic acid, vitamin B12, zinc, copper, and magnesium levels • CSF: mildly elevated WBC count with lymphocytic predominance (10, normal 0–8/ul), normal protein (20 mg/dl), negative MS and encephalopathy panels, negative VZV CSF IgG • EMG/NCS: unremarkable for either neuropathy or radiculopathy with decreased activation indicative of a central cause of weakness	• Normal CBC and electrolytes
Neuro-imaging findings	• CT head: Bilateral hypoattenuation within paramedian parietal and occipital lobes • CTA head and neck: Areas of mild-to-moderate multifocal stenosis of bilateral MCAs, PCAs, and ACAs	• CT head: Cortical SAH in right temporal and parietal lobes. • CTA head and neck: No intracranial aneurysm, vascular malformation, or vasospasm. • DSA: Diffuse narrowing and irregularities in bilateral distal MCAs’ cortical branches that improved after intraarterial verapamil
Management	• Verapamil 120 mg QHS for CVS, and discharged to an AR facility	• Verapamil 60 mg BID for CVS, and discharged home with good recovery
RCVS_2_ score	• 6 (Thunderclap headache [5], female gender [1])	• 7 (Thunderclap headache [5], female gender [1], SAH [1])

Abbreviations: MRC; Medical Research Council, CBC; Complete blood count, CSF; Cerebrospinal fluid, WBC; White blood cell, MS; Multiple sclerosis, VZV; Varicella-Zoster virus, EMG/NCS; Electromyography/Nerve conduction study, CT; Computed tomography, CTA; CT angiography, DSA; Digital subtraction angiography, MCAs; middle cerebral arteries, PCAs; Posterior cerebral arteries, ACAs; Anterior cerebral arteries, SAH; Subarachnoid hemorrhage, CVS; Cerebral vasospasm, RCVS; Reversible cerebral vasoconstriction syndrome, BID; Twice daily, QHS; Nightly, AR; Acute rehabilitation.

NB: The RCVS_2_ score was adapted from this reference: **Rocha EA, Topcuoglu MA, Silva GS, Singhal AB. RCVS2 score and diagnostic approach for reversible cerebral vasoconstriction syndrome. Neurology. 2019 Feb 12;92(7):e639-e647. doi:**  **10.1212/WNL.0000000000006917. Epub 2019 Jan 11. PMID: 30635475** (https://pubmed.ncbi.nlm.nih.gov/30635475/). A score of ≥ 5 had 99% specificity and 90% sensitivity for diagnosing RCVS.

Given the temporal association between starting teprotumumab and patient’s headaches with subsequent neurological symptoms, the presence of radiologic features of reversible CVS, and the absence of serum and CSF inflammatory markers suggestive of a vasculitic process, patient’s presentation was most consistent with RCVS, and teprotumumab is suggested to be the potential trigger factor.

### Case 2

A 71-year-old female with history of GED for which she was started on teprotumumab two months earlier, was admitted to our institution after experiencing a sudden onset of thunderclap headache associated with nausea and vomiting, and no additional neurological complaints. The neurological examination was otherwise unremarkable.

**Figure 2:**
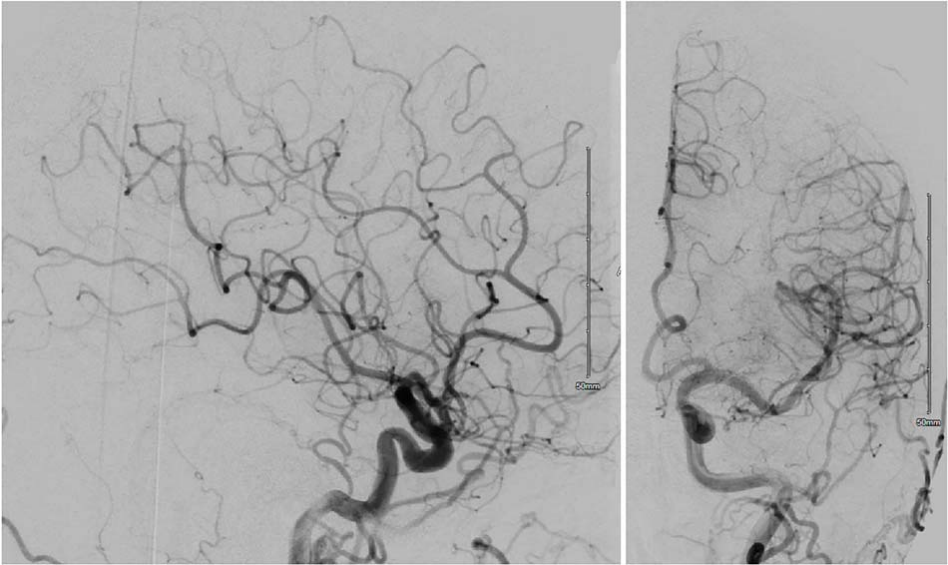
DSA of Case 2 illustrating distal segmental narrowing of MCA cortical branches.

Laboratory work-up showed normal CBC and electrolytes. CT head showed cortical SAH in the right temporal and parietal lobes. CTA head and neck showed no intracranial aneurysm, vascular malformation, or CVS. However, digital subtraction angiography (DSA) showed diffuse narrowing and irregularities in bilateral distal MCAs’ cortical branches that improved following intraarterial verapamil administration (Fig. 2). Thus, the patient was started on verapamil 60 mg twice daily for the management of CVS. She improved clinically and was discharged home with no residual neurological deficits (Table 1).

Considering the abrupt onset of a thunderclap headache, the radiological features indicative of CVS, and the temporal correlation between starting teprotumumab and symptom onset, the clinical presentation aligned most likely with RCVS, with teprotumumab suggested as the potential triggering factor.

## Discussion

The exact pathophysiology of RCVS is still unclear despite identifying multiple risk factors including several medications from different classes and a few medical conditions such as pregnancy, cervical artery dissection [3], and metabolic derangements [4]. However, disruption of cerebral autoregulation and dysfunction of the endothelial lining of the cerebral vasculature are considered the cornerstone of RCVS’s pathophysiology as evidenced by comparative case–control studies. The cerebrovascular endothelial dysfunction occurs through variable mechanisms according to the underlaying etiology [3].

IGF-1 has neuroprotective effects against cerebral ischemic insults [8]. Animal studies suggested that IGF-1 deficiency impairs the autoregulation of cerebral vasculature via multiple mechanisms including endothelial dysfunction, disrupting cerebral blood flow responses, decreasing the elastin content in the cerebral vasculature [9], and compromising the nitric oxide release from endothelial cells [10]. Furthermore, a few studies found that lower IGF-1 levels are associated with increased risk of ischemic stroke, and higher IGF-1 levels after ischemic strokes are associated with better functional recovery [9]. Overall, these findings suggest that alterations of IGF-1 levels and/or function might be associated with both rapid and long-term effects on the cerebral vasculature.

Teprotumumab acts by antagonizing IGF-1R and is used for the treatment of GED since IGF-1R is upregulated in orbital fibroblasts and immune cells in GED patients [5]. Given the aforementioned involvement of IGF-1 in cerebrovascular modulation, we hypothesize that teprotumumab is the etiological factor triggering RCVS in the two cases we are reporting. However, with scarce reporting on this complication, it is still unclear whether the assumed etiology of RCVS in those patients is solid.

### Key points

RCVS is a potential adverse effect of teprotumumab, and further research is needed to confirm this association. If a causal association is confirmed, it is necessary to investigate if there are risk factors that might increase the risk of RCVS development in certain patients receiving teprotumumab rather than the others. Moreover, close monitoring will be needed for patients receiving teprotumumab for GED.

## Conclusion

RCVS is a rare clinical condition that might be associated with serious sequalae if not addressed properly and timely. Our report indicates possible involvement of teprotumumab in triggering RCVS, and the hypothesized pathophysiology is via antagonizing IGF-1’s neuroprotective effects. Further research is needed to corroborate these observations.

## CRediT statement

Mohamed Elfil: contributed by obtaining consents, drafting, and preparing the manuscript for submission.

Pashayar P Lookian: contributed by drafting and preparing the manuscript for submission.

Kanchan Kumari: contributed by obtaining consents and preparing the manuscript for submission.

Mohammad Aladawi: contributed by designing the figures, drafting, and preparing the manuscript for submission.

Mark Jedras: contributed by preparing the manuscript for submission.

Steven M Phillips: contributed by conceptualizing and revising the manuscript for submission.

Mithun G Sattur: contributed by conceptualizing and revising the manuscript for submission.
